# Synergistic Biomedical Potential and Molecular Docking Analyses of Coumarin–Triazole Hybrids as Tyrosinase Inhibitors: Design, Synthesis, In Vitro Profiling, and In Silico Studies

**DOI:** 10.3390/ph17040532

**Published:** 2024-04-20

**Authors:** Rukhsana Kausar, Ameer Fawad Zahoor, Hina Tabassum, Shagufta Kamal, Mashooq Ahmad Bhat

**Affiliations:** 1 Department of Chemistry, Government College University Faisalabad, Faisalabad 38000, Pakistan; rukh8517@gmail.com; 2 Department of Pharmacology, London Metropolitan University, 166-220 Holloway Road, London N7 8DB, UK; 3 Department of Biochemistry, Government College University Faisalabad, Faisalabad 38000, Pakistan; shaguftakamal81@gmail.com; 4 Department of Pharmaceutical Chemistry, College of Pharmacy, King Saud University, Riyadh 11451, Saudi Arabia

**Keywords:** coumarin, triazoles, tyrosinase inhibitors, synthesis

## Abstract

The tyrosinase enzyme has a vital role in the browning of vegetables and fruits and the biosynthesis of melanin. In this work, we synthesized a diverse library of coumarin–triazole hybrids, and these compounds were characterized by using suitable analytical techniques. Our research work extends beyond the synthetic effort to explore the therapeutic potential of these compounds. We put the synthesized compounds through meticulous in vitro screening against the tyrosinase enzyme, and these coumarin derivatives evinced good IC_50_ values in the range of 0.339 ± 0.25 µM to 14.06 ± 0.92 µM. In the library of synthesized compounds, six compounds were found to be more potent than standard ascorbic acid (IC_50_ = 11.5 ± 1.00), and among them, **17e** and **17f**, being the most active, exhibited remarkable anti-tyrosinase potential, with IC_50_ values of 0.339 ± 0.25 μM and 3.148 ± 0.23 μM, respectively. Furthermore, an in silico modeling study was carried out to determine the key interactions of these compounds with the tyrosinase protein (PDB ID: 2Y9X) and thus to authenticate our experimental findings. The quantitative SAR studies exhibited a good correlation between the synthesized derivatives of coumarin and their anti-tyrosinase activity. The docking studies verified the experimental results, and ligand **17e** showed good interaction with the core residues of tyrosinase. This study not only expands the field of coumarin–triazole hybrid synthesis but also provides valuable insights for the development of novel tyrosinase inhibitors.

## 1. Introduction

Tyrosine, a precursor for the biosynthesis of catecholamines, including tyramine, L-DOPA, and dopamine, is oxidized to dopaquinone in mammalian melanogenesis by the copper-containing enzyme tyrosinase [1,2,3]. Melanogenesis in skin is a requisite for protection against UV radiation, which causes skin cancer, while an excessive amount of melanin can cause diseases like cancer, solar lentigo, ephelis, age spots, melanoderma, and malignant melanoma [4]. In addition, tyrosinase catalyzes the synthesis of neuromelanin in the brain and other polyphenolic compounds in the hair and skin [5]. Moreover, tyrosine is responsible for the process aimed at perpetuating the flavor, nutritional value, appearance, and texture of many fruits. Both elevated and low tyrosine levels in biological fluids are associated with many metabolic disorders. The browning of fruits and fungi after the harvest of crops such as mushrooms and that of beverages are also due to an abnormal level of tyrosine in plants [6]. Women are vulnerable to dermatological disorders such as freckles, senile lentigo, pigmented acne scars, and melasma, and these hyper-pigmentation conditions are directly associated with hyper-accumulation of melanin [7].

**Figure 1:**
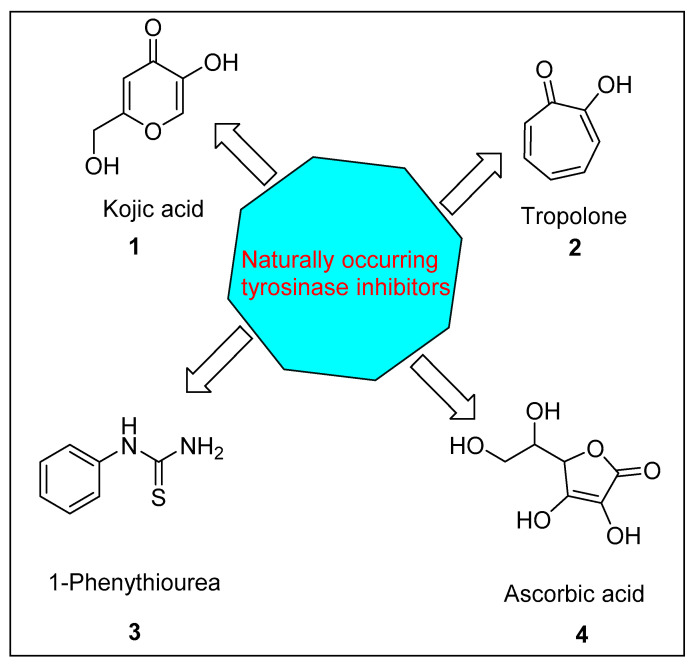
Naturally occurring tyrosinase inhibitors (**1**–**4**).

Compounds with inhibition activity for tyrosinase have been used for the treatment of hyper-pigmentation of the skin and the enzymatic browning of fruits [8]. Neurodegenerative diseases including Parkinson’s are also related to a high level of tyrosine in body fluids [9]. It has been found that tyrosinase is linked with different biochemical processes taking place in insects, such as defensive encapsulation and the sclerotization of cuticles [10]. Taking into consideration the importance of tyrosinase inhibitors, medicinal chemists are engaged in an abiding search for novel, effective, and safe tyrosinase inhibitors. The synthesis of an efficient and effective arsenal of compounds (anti-tyrosinase) is of eminent concern in the agricultural, medical, and cosmetic industries [11]. Many natural compounds have been found as potent tyrosinase inhibitors; for example, flavonoid derivatives are reported as the strongest inhibitors of tyrosinase [12]. Kojic acid (**1**), tropolone (**2**), ascorbic acid (**4**), and 1-phenylurea (**3**), depicted in Figure 1, are naturally occurring compounds with potent tyrosinase inhibition activity [13]. However, these natural drugs still have unpleasant side-effects, such as dermatitis, cutaneous melanoma, and cytotoxicity. These problems lead us to design more active and safer tyrosinase inhibitors [14]. Heterocycles such as coumarins [15], quinoxalines [16], oxadiazoles [17,18,19], acefylline [20], triazoles [21], lamivudines [22], thiadiazoles [23,24], quinoxaline-sulfonamides [25], ciprofloxacin-oxadiazoles [26], etc., show a variety of biological activities against different diseases.

**Figure 2:**
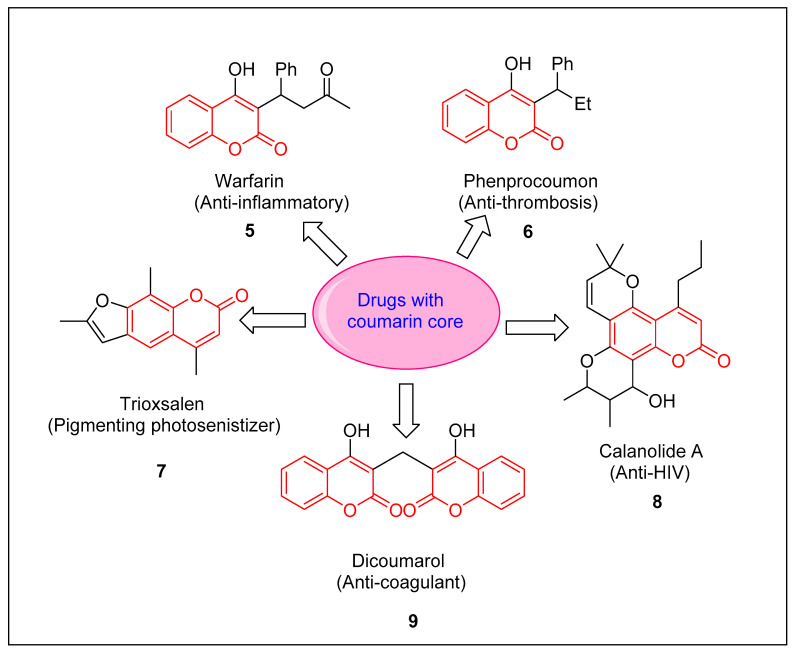
Some commonly used drugs having coumarin scaffolds.

Coumarin derivatives are captivating compounds due to the presence of coumarin (as a structural motif) in many natural drugs with interesting photophysical properties and biological applications [27,28]. In addition to having anti-oxidant and anti-microbial activities [29], coumarins are considered to represent an important class of organic compounds amenable to act as anti-cancer agents [30]. Coumarins are also very familiar as anti-HIV [31], anti-coagulant [32], and immunosuppressive drugs [33]. Some coumarin-based drugs are depicted in Figure 2. In addition to their pharmacological uses, molecules with a coumarin core have also been found to be used as luminescent probes, food additives, and dyes [34]. Matos et al. synthesized dihydroxy thiophene substituted coumarin as a potent anti-tyrosinase agent with an IC_50_ = 0.19 µM [35]. As a consequence of coumarin’s applications in various fields, modifications of this scaffold have been profoundly researched. A number of coumarin scaffolds (**5**–**9**) commonly employed as drugs are presented in Figure 2.

Triazole analogs are considered a large array of medicinally valuable compounds [36] owing to their anti-tubercular, anti-microbial, anti-viral, and anti-cancer activities [37,38]. Particularly, many compounds with a 1,2,4-triazole moiety are found to be potent inhibitors of tyrosinase [39,40,41,42,43,44]. Coumarins and triazoles are structural motifs that show tremendous potential biological activity due to their high therapeutic indexes [45,46]. Since both triazoles and lactones are biologically active pharmacophores, novel molecules with these bioactive moieties have been found to be more effective drugs than the parent compounds with a single moiety. For instance, the triazole–coumarin hybrids exhibit potential nonpeptidic [47], protease transglutaminase [48], glycogen phosphorylase, and glucose-6-phosphatase [49] inhibition activities.

**Figure 3:**
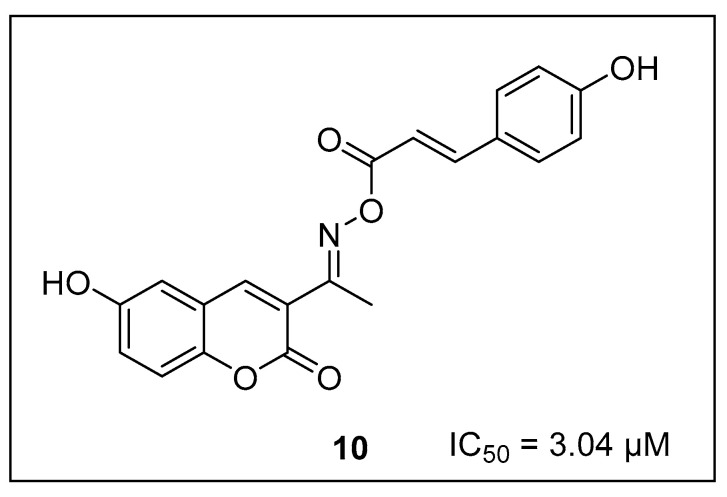
Potent tyrosinase inhibitor with coumarin core.

Some of the coumarin derivatives also exhibit inhibition activity against the tyrosinase enzyme [50,51,52]. According to Li and coworkers (2023), 2-(1-(coumarin-3-yl)ethylidene)hydrazine carbothio-amide (**10**) (Figure 3) is a potent tyrosinase inhibitor with an IC_50_ of 3.04 µM [53].

So far, the best possible drug for tyrosinase inhibition is yet to be synthesized, and in this regard, a meticulous search for an innovative tyrosinase inhibitor is still required. Molecular hybridization, which has been considered an important strategy to design novel drugs, combines two or more biologically active moieties to synthesize a new pharmacological hybrid molecule [54].

Molecular hybridization is the main strategy to enhance the biological potential of heterocycles and can possibly overcome the challenge of drug resistance. It has been observed that the incorporation of a triazole ring with different biologically active moieties imparts potent biological properties to the molecules. Bearing in mind this observation, we designed and synthesized novel coumarin–triazole hybrids in the present research work. These synthetic compounds were assayed for their tyrosinase inhibition activity, which was further confirmed by an in silico induced fit docking (IFD) simulation.

## 2. Result and Discussion

### 2.1. Synthesis

**Scheme 1:**
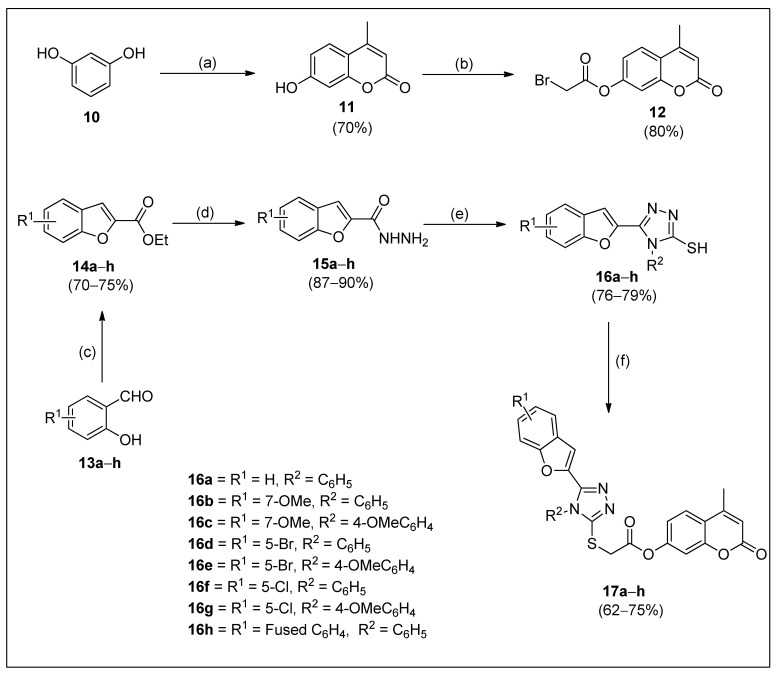
Synthetic strategy for the synthesis of coumarin derivatives (**17a**–**h**). Reagents and conditions: (a) Ethyl acetoacetate and H_2_SO_4_; 25 °C. (b) 2-Bromoacetyl bromide, pyridine, and CHCl_3_; r.t. (c) Ethyl chloroacetate, DMF, and K_2_CO_3_; 90–95 °C. (d) Hydrazine hydrate and methanol; r.t. (e) NaOH, and H_2_O; reflux. (f) **12**, K_2_CO_3_, and DCM; r.t.

The synthetic process of the target coumarin–triazole hybrids is depicted in Scheme 1. Briefly, to probe the impact of substitution in the aromatic part of coumarin on tyrosinase inhibitory activity, the compound 7-hydroxy-4-methylcoumarin (**11**; used as a precursor for further synthetic manipulation) was synthesized (70%) by the Pechmann condensation of commercially available resorcinol (**10**) with ethylacetoacetate by using a catalytic amount of sulfuric acid, followed by its reaction with 2-bromoacetylbromide in the presence of pyridine to afford 4-methyl-2-oxo-2H-chromen-7-yl carbonobromidate (**12**) (80%). Different substituted *o*-hydoxybenzaldehydes (**13a**–**h**) were treated with ethyl chloroacetate in the presence of K_2_CO_3_ to procure a variety of ethyl benzofuran-2-carboxylates (**14a**–**h**) (70–75%). Afterwards, substituted benzofuran-2-carbohydrazides (**15a**–**h**) (87–90%) were obtained by treating the ethyl benzofuran-2-carboxylates (**14a**–**h**) with hydrazine hydrate in MeOH. These substituted benzofuran-2-carbohydrazides (**15a**–**h**) were converted into thiosemicarbazides by using arylisothiocyanates. Various 1,2,4-triazoles (**16a**–**h**) (76–79%) were afforded by the cyclization of substituted 2-(benzofuran-2-carbonyl)hydrazine-1-sulfinimidamides in the presence of aqueous NaOH solution. To furnish the targeted coumarin–triazole hybrids (**17a**–**h**), K_2_CO_3_ was added to the solution of substituted 5-(benzofuran-2-yl)-4H-1,2,4-triazole-3-thiols (**16a**–**h**) in DCM (dichloromethane). Then, 4-methyl-2-oxo-2H-chromen-7-yl carbonobromidate (**12**) was added, and the reaction mixture was stirred to obtain the target compounds (**17a**–**h**) in a 62–75% yield range.

### 2.2. Spectral Description of the Most Potent Compound (***17e***)

4-Methyl-2-oxo-2H-chromen-7-yl-2-((5-(5-bromobenzofuran-2-yl)-4-(4-methoxyphenyl)-4H-1,2,4-triazol-3-yl)thio)acetate was synthesized as a coarse off-white solid. Its molecular formula/purity was ascertained by elemental analysis, and structural elucidation was attained through ^1^H and ^13^C NMR spectroscopy.

**Figure 4:**
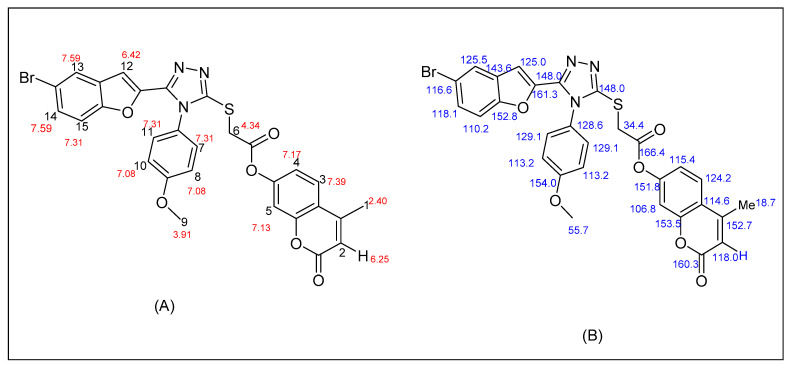
^1^HNMR (**A**) and ^13^CNMR (**B**) interpretations of representative derivative **17e**.

In the ^1^H-NMR spectrum, at δ 4.34, the signal for H-6 between S and carbonyl group was observed. Peaks for three protons of methyl groups at H-1 and H-9 were detected at δ 2.40 and δ 3.91, respectively. 2H-8 and 2H-10 resonated at δ 7.08, whereas 2H-7 and 2H-11 of the phenyl ring resonated at δ 7.31 as a singlet. Aromatic protons H-14 and H-13 reverberated as a doublet at δ 7.59 with a coupling constant (*J*) of 8 Hz, while proton-14 and proton-2 appeared at δ 6.42 and δ 6.25, respectively (Figure 4). The carbon scaffold of the aforesaid compound was also confirmed by ^13^C NMR. Signals for all the 20 carbons were detected in the spectrum, and the upfield signal at δ 34.4 confirmed the development of the methylene linker between compounds **11** and **15**. The other two signals of carbons at δ 148 and δ 148 authenticated the presence of a 1,2,4-triazole ring. The substituted phenyl carbons showed their signals at δ 113, δ 128, and δ 129. In the same way, signal for carbons of the benzofuran ring appeared at δ 110, δ 116, δ 118, δ 125, δ 144, δ 152, and δ 161 (Figure 4). The benzofuran and the benzene rings in compound **17e** bore the electron-donating groups Br and OMe, respectively. The spectroscopic studies confirmed the structure of **17e**. Other synthetic derivatives were structurally characterized by a similar approach (Figures S1–S16).

### 2.3. Anti-Tyrosinase Activity of Target Compounds

All the synthesized triazole derivatives of coumarin exhibited tyrosinase inhibition activity comparable to that of the standard inhibitor, ascorbic acid (IC_50_ = 11.5 ± 1.00), with IC_50_ (half maximal inhibitory concentration) values ranging from 0.33 ± 0.08 µM to 14.06 ± 0.17 µM, as given in Table 1.

Compounds **17e**, **17f**, and **17g** exhibited excellent potent activity, with IC_50_ of 0.339 ± 0.08, 3.148 ± 0.23, and 3.982 ± 0.12, respectively, while **17b** and **17c** showed comparatively lower enzyme inhibition activity. The substitution and position on the benzene ring of the benzofuran nucleus significantly increases or decreases the potent inhibition activity of the synthesized scaffolds **17a**–**h**.

### 2.4. Structure–Activity Relationship (SAR) of Potent Anti-Tyrosinase Derivatives

The SAR (structure–activity relationship) interpretation of the present findings revealed that the halo substitution on the benzene ring manifests the promising inhibitory effects of these derivatives. Among all the synthesized triazole derivatives of coumarin, scaffold **17e** with a bromine group at the benzofuran ring showed the best inhibition activity, with an IC_50_ = 0.339 ± 0.08 µM, which is even much higher than those of the reference inhibitory drugs, ascorbic acid (IC_50_ = 11.5 ± 1.0 µM) and kojic acid (IC_50_ = 30.34 ± 0.75 µM). The IC_50_ values of **17f** and **17g** with Cl-substituted aromatic rings were observed to be 3.148 ± 0.23 µM (%age inhibition of 36.134 ± 0.21) and 3.982 ± 0.12 µM (31.0609 ± 0.19), respectively, which are also greater than those of the frequently used standards. In a similar way, high enzyme inhibition activity was displayed by **17d** (IC_50_ = 5.893 ± 0.09 and %age inhibition = 23.097 ± 0.13), which bears a Br-substituted aromatic ring. Moderate tyrosinase inhibitory potential (IC_50_ = 8.138 ± 0.13 and %age inhibition = 12.023 ± 0.21) was exhibited by **17a**, which carries an unsubstituted benzofuran ring. On the other hand, compounds **17b** (%age inhibition = 6.3009 ± 0.14 and IC_50_ = 14.062 ± 0.17) and **17c** (%age inhibition = 5.110 ± 0.15 and IC_50_ =12.987 ± 0.32) with methoxy benzofuran substitution proved to be less active against the tested enzyme concentration. The comparison of the IC_50_ values of all compounds showed that not substituting and substituting the benzofuran ring with electron-donating groups such as OMe lowered the tyrosinase inhibition activity.

### 2.5. In Silico Modeling of Representative Potent Tyrosinase Inhibitor (***17e***)

**Figure 5:**
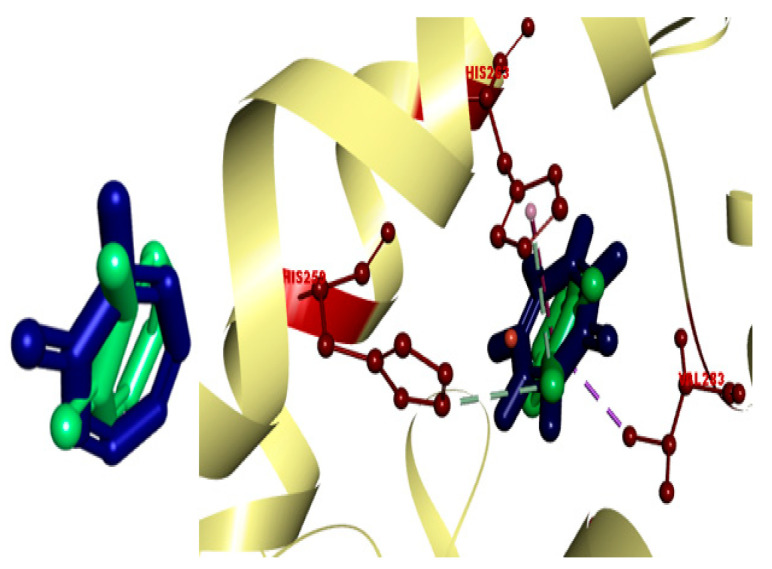
Induced fit docking between redocked (blue) and native ligand (green) validated by RMSD.

The most potent coumarin–triazole hybrids, **17e** and **17f**, were subjected to molecular docking (IFD docking) to elaborate the vital interactions which are liable for the binding between ligand and protein. The binding energies and binding modes of the aforesaid compounds were also investigated. We used the 2Y9X (protein database code) crystal structure, in which the enzyme tyrosinase belongs to the same species of mushroom, *A. bisporus*, which was used in in vitro experimental studies. Tyrosinase (2Y9X) is a tetramer in which tropolone is bound to various subunits, thus having different conformation depending upon the subunits. Through cognate redocking, the dependability of the docking study was assessed. In MOE, the native tropolone ligand was redocked. To validate our docking protocol, the RMSD was calculated between the atomic coordinates of the co-crystallized and theoretical poses of the tropolone inhibitor that was present in the crystal structure (2Y9X). The self-docking result revealed a root mean square deviation (RMSD) of less than 1.5 Å between the native and redocked ligands (Figure 5).

A pyran ring with an oxo group is a common constituent in the structures of Kojic acid and most potent compounds. However, Kojic acid has hydroxyl and hydroxy methyl groups attached to the pyran ring, while **17e** and **17f** have a triazole ring and a benzofuran ring, along with a coumarin core. The binding interactions of the selected compounds, **17e** and **17f**, with the receptor site of the protein (2Y9X) were calculated. Both of the compounds showed higher binding interactions, −6.75 kcal/mol for **17e** and −6.29 kcal/mol for **17f**, respectively, compared with the standard IFD threshold for Kojic acid (∆G −5.25 kcal/mol), indicating a better potential of these compounds (Table 2).

**Figure 6:**
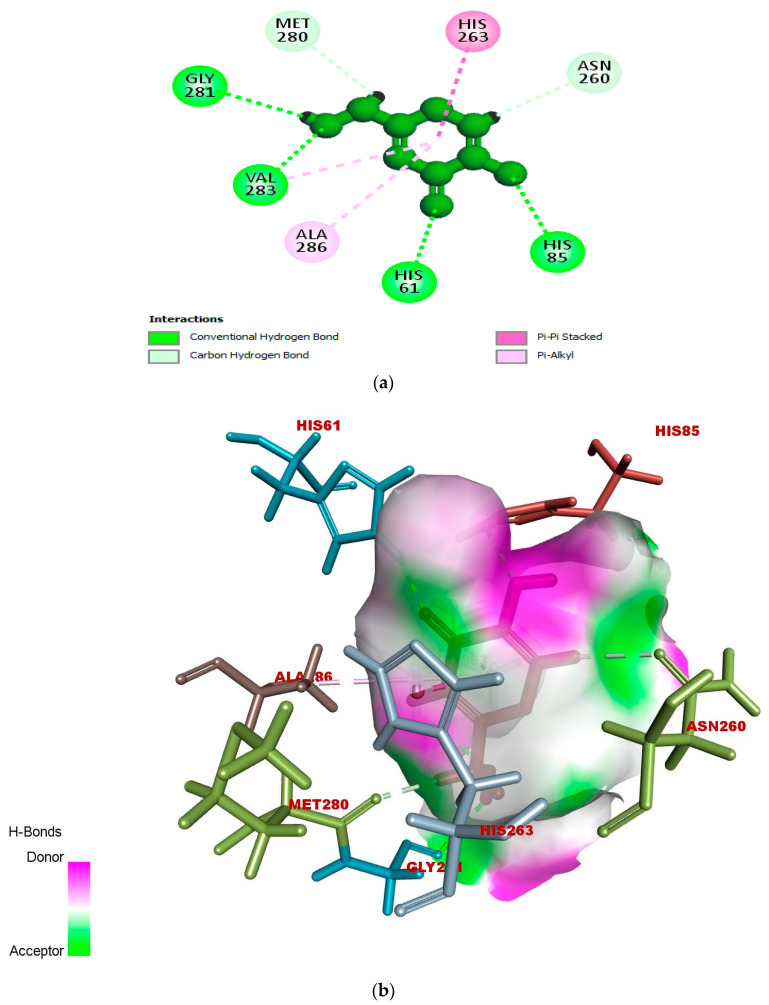

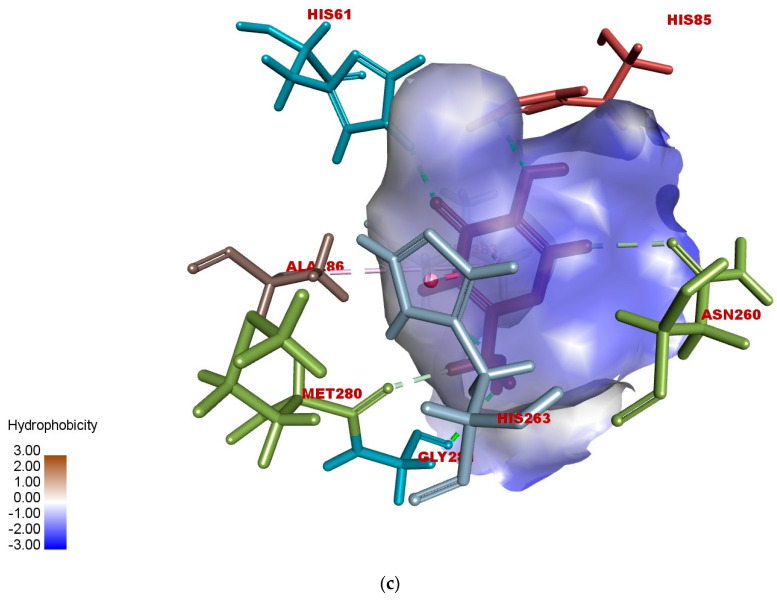
(**a**) Two-dimensional interaction of standard Kojic acid with binding pocket of 2Y9X. (**b**) Three-dimensional ligand–protein hydrogen-bonding interactions of standard Kojic acid. (**c**) Three-dimensional ligand–protein hydrogen-bonding interactions of standard Kojic acid.

**Figure 7:**
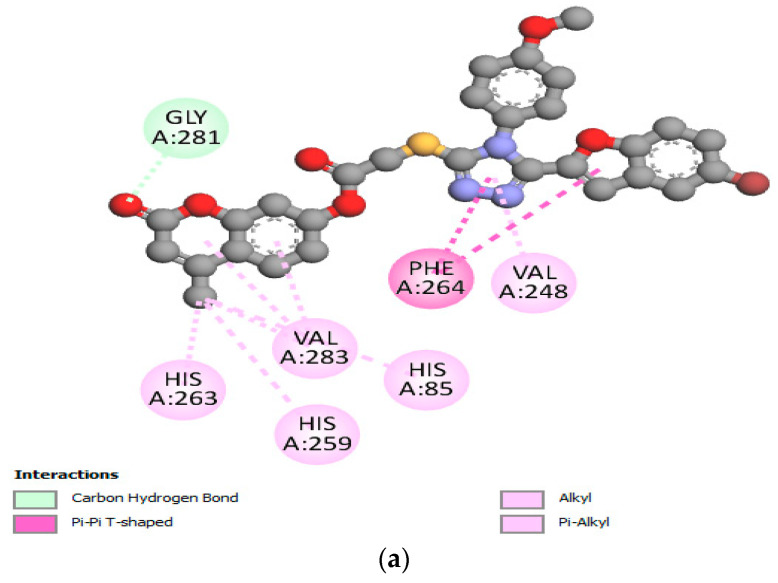

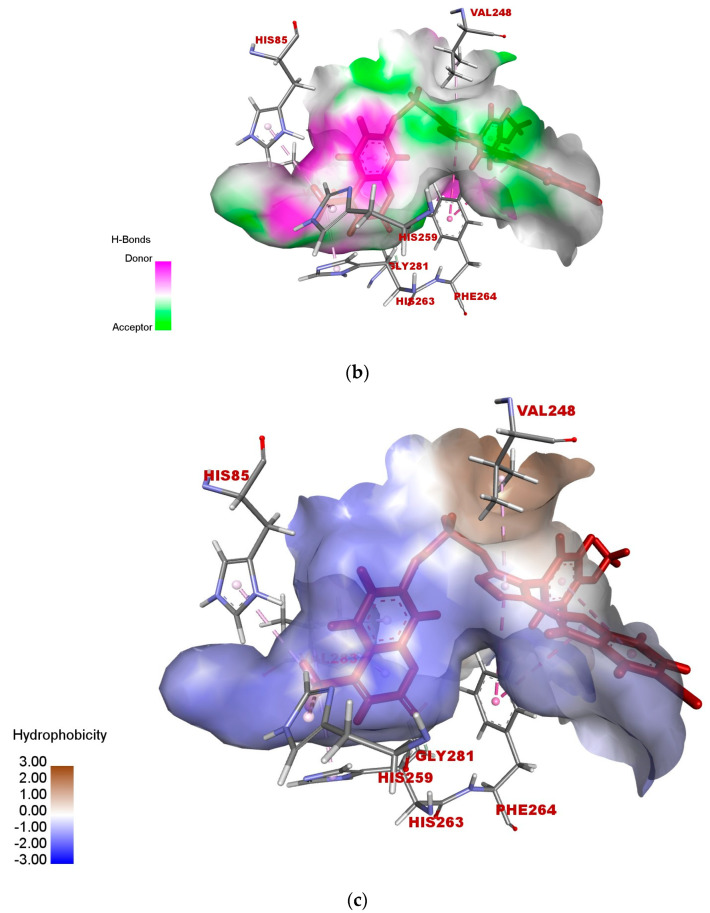
(**a**) Two-dimensional interaction of anti-tyrosinase agent **17e** with active site of 2Y9X. (**b**) Docking pose of potent anti-tyrosinase agent **17e** at active site of tropolone hydrophobic cavity of 2Y9X showing hydrogen-bonding interactions. (**c**) Docking pose of potent anti-tyrosinase agent **17e** at active site of tropolone hydrophobic cavity of 2Y9X showing hydrophobic interactions.

**Figure 8:**
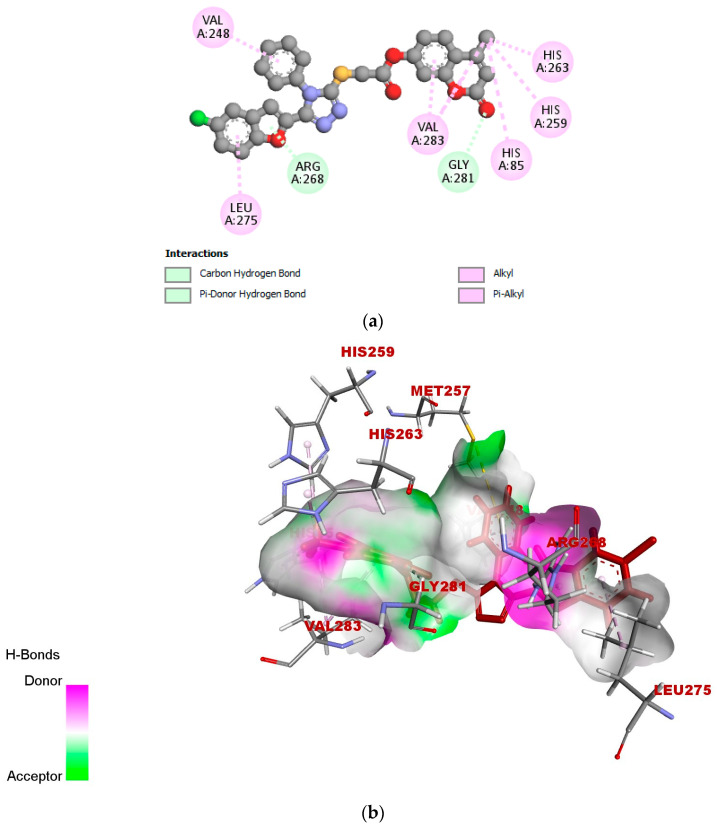

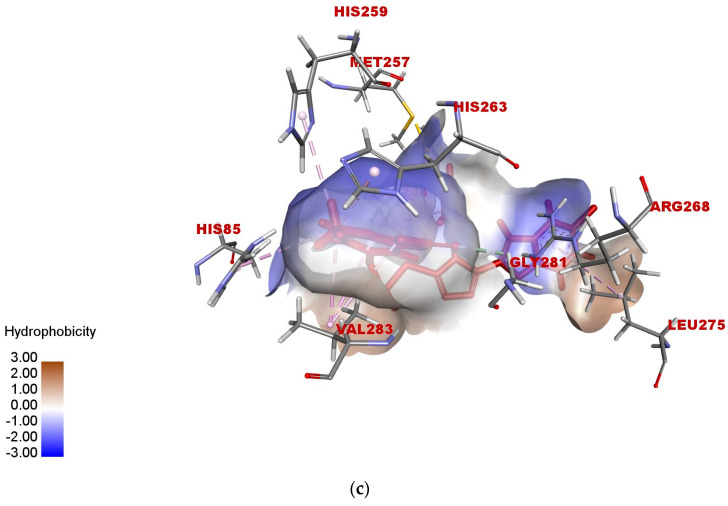
(**a**) Two-dimensional interaction diagram of compound **17f** with active site of tyrosinase (2Y9X). (**b**) Hydrogen-bonding interactions of **17f** with binding sites of 2Y9X. (**c**) Hydrophobic interactions of **17f** with binding sites of 2Y9X.

According to the docked compound’s ligand–protein binding interaction study, they engage with the receptor site surrounding the copper vicinity, which could possibly be the cause of the inhibition of tyrosinase. Kojic acid was found to have prominent hydrogen-bonding (GLY-281, HIS-61, VAL-283, HIS-85, MET-280, and ASN-260) and hydrophobic (ALA-286 and HIS-263) interactions (Figure 6a–c). In the same way, compound **17e** displayed hydrophobic interactions, as well as hydrogen-bonding interactions with residues. In detail, **17e** showed strong bonding with GLY-281 in carbon–hydrogen bond interactions at a distance of 2.55 Å, and PHE-264 formed π hydrophobic interactions with the triazole ring and furan π-system at distances of 5.27 and 5.46 Å, respectively (Figure 7a–c). The furan π-system also interacted with VAL-248 (4.36 Å). Similarly, the methyl group on the coumarin ring displayed an alkyl hydrophobic interaction with VAL-283 (4.19 Å), HIS-263 (3.67 Å), HIS-259 (4.82 Å), and HIS-85 (5.04 Å). VAL-283 also showed a π hydrophobic interaction with the coumarin π-system at distances of 4.55 Å and 4.51 Å. Furthermore, compound **17f** displayed hydrogen bonding with GlY-281 and ARG-268 at distances of 2.59 and 2.61 Å, respectively (Figure 8a–c). LEU-275 and VAL-248 formed π hydrophobic interactions with the coumarin and phenyl group of triazole at distances of 5.21 Å and 4.04 Å, respectively. Similarly, the methyl group of **17f** formed alkyl hydrophobic interactions with VAL-283 (4.52Å), HIS-259 (4.77 Å), HIS-85 (5.13 Å), and HIS-263 (3.57 Å), as well as a π hydrophobic interaction with VAL-283 at distances of 4.31 Å and 4.29 Å. Additionally, the hydroxylpyranone part of Kojic acid can chelate with the Cu^2+^ at the active site of tyrosinase, while the coumarin core of compound **17e** seemed to be involved in a *π*-stacking interaction with the His-263 residue, which makes a complex with one of the Cu^2+^ ions. 

Therefore, the experimental results, identifying the aforesaid compounds (**17e** and **17f**) as strong tyrosinase inhibitors, were corroborated by the induced fit docking analysis.

## 3. Materials and Methods

### 3.1. Chemicals and Reagents

Kojic acid and ascorbic acid were obtained from Sigma–Aldrich (St. Louis, MO, USA), while the other required chemicals were bought from local suppliers. All chemicals were purified and dried before use. A WRS-1B mp apparatus was used for melting point determination of the synthesized compounds, and the thermometer was uncorrected. For drying organic phases, anhydrous Na_2_SO_4_ was used. At 25 °C, all NMR spectra were recorded by using a Bruker 400 spectrometer (Bruker, Zurich, Switzerland) in DMSO-d_6_ or CDCl_3_, and the δ (chemical shifts) values were reported in ppm downfield from internal tetramethylsilane (TMS). *J* (coupling constants) are given in Hz, and the abbreviations used for the splitting patterns are s for singlet, d for doublet, t for triplet, and m for multiplet. Progress of all reaction processes was monitored by analytical technique TLC (thin-layer chromatography) using the *n*-hexane-to-ethyl acetate ratio, while spots were visualized under ultraviolet light.

### 3.2. General Synthetic Protocol for Synthesis of Substituted 4-Methyl-2-oxo-2H-chromen-7-yl-2-((5-(benzofuran-2-yl)-4H-1,2,4-triazol-3-yl)thio)acetates (***17a**–**h***)

In a solution of substituted benzofuran-based triazoles (**16a**–**h**) (1 mmol) in DCM (dichloromethane) (6 mL), K_2_CO_3_ (1.2 mmol) and 4-methyl-2-oxo-2H-chromen-7-yl carbonobromidate (**12**) (1.1 mmol) were added. The reaction mixture was allowed to stir at 25 °C and monitored by TLC, which indicated reaction completion after 16 h. Precipitates of the desired products (**17a**–**h**) (62–75%) were obtained upon the addition of n-hexane and distilled water. The crude products were purified via recrystallization by using ethanol.

#### 3.2.1. 4-Methyl-2-oxo-2H-chromen-7-yl-2-((5-(benzofuran-2-yl)-4-phenyl-4H-1,2,4-triazol-3-yl)thio)acetate (**17a**)

Coarse off-white solid; yield: 65%; mp 152 °C; ^1^H NMR (CDCl_3_, 400 MHz) δ (ppm): 2.40 (s, 3H); 4.32 (s, 2H); 6.25 (s, 1H); 6.55 (s, 1H); 7.13 (s, 1H); 7.18 (d, *J* = 8 Hz, 1H); 7.27 (s, 1H); 7.35 (s, 1H); 7.37–7.42 (m, 3H); 7.54–7.68 (m, 6H). ^13^C NMR (CDCl_3_, 100 MHz): δ (ppm) 18.7, 34.5, 107.5, 110.2, 113.2, 114.6, 116.7, 118.0, 118.1, 124.4, 125.5, 125.6, 127.3, 127.3, 129.1, 129.28, 130.3, 130.3, 131.1, 132.7, 143.2, 147.5, 151.8, 152.8, 153.5, 154.0, 160.3, 166.3. MS *m*/*z*: 509.9 [M]^+^. Anal. Elem. Calc. for C_28_H_19_N_3_O_5_ S: C, 66.00: H, 3.76: N, 8.25: Found: C, 66.02: H, 3.78: N, 8.24.

#### 3.2.2. 4-Methyl-2-oxo-2H-chromen-7-yl-2-((5-(7-methoxybenzofuran-2-yl)-4-phenyl-4H-1,2,4-triazol-3-yl)thio)acetate (**17b**)

Amorphous white solid; yield: 75%; mp 140 °C; ^1^H NMR (CDCl_3_, 400 MHz) δ (ppm): 2.40 (s, 3H); 3.81 (s, 3H); 4.34 (s, 2H); 6.25 (s, 1H); 6.77 (t, *J* = 20 Hz 2H); 7.05–7.12 (m, 3H); 7.17 (d, *J* = 12 Hz 1H); 7.41 (d, *J* = 8 Hz 2H); 7.57–7.62 (m, 4H). ^13^C NMR (CDCl_3_, 100 MHz): δ (ppm) 18.7, 34.5, 56.4, 108.6, 109.3, 109.4, 110.2, 114.6, 118.0, 118.1, 124.3, 125.5, 127.4, 127.5, 129.0, 130.0, 130.1, 130.6, 130.7, 133.1, 142.2, 144.5, 145.5, 148.0, 151.9, 152.8, 154.0, 160.4, 166.4. MS *m*/*z*: calcd. 539.9 [M]^+^. Anal. Elem. Calc. for C_29_H_21_N_3_O_6_S: C, 64.56: H, 3.92: N, 7.79: Found: C, 64.53: H, 3.93: N, 7.80.

#### 3.2.3. 4-Methyl-2-oxo-2H-chromen-7-yl-2-((5-(7-methoxybenzofuran-2-yl)-4-(4-methoxyphenyl)-4H-1,2,4-triazol-3-yl)thio)acetate (**17c**)

Coarse off-white powder; yield, 64%; mp 137 °C; ^1^H NMR (CDCl_3_, 400 MHz) δ (ppm): 2.36 (s, 3H); 3.82 (s, 3H); 3.85 (s, 3H); 4.27 (s, 2H); 6.2 (s, 1H); 6.57 (s, 1H); 6.74 (d, *J* = 8 Hz, 1H); 7.0–7.08 (m, 5H); 7.13 (d, *J* = 8 Hz, 1H); 7.26 (d, *J* = 8 Hz, 2H); 7.54 (d, *J* = 8 Hz, 1H). ^13^C NMR (CDCl_3_, 100 MHz): δ (ppm) 18.7, 34.4, 55.7, 56.4, 108.2, 109.0, 110.2, 113.8, 114.6, 114.6, 115.2, 115.3, 118.0, 124.2, 125.5, 125.5, 128.6, 128.6, 129.1, 142.5, 144.5, 145.5, 148.3, 151.9, 152.1, 152.8, 154.0, 160.4, 161.1, 166.5. MS *m*/*z*: 569.9 [M]^+^. Anal. Elem. Calc. for C_30_H_23_N_3_O_7_S: C, 63.26: H, 4.07: N, 7.38: Found: C, 63.25: H, 4.09: N, 7.39.

#### 3.2.4. 4-Methyl-2-oxo-2H-chromen-7-yl-2-((5-(5-bromobenzofuran-2-yl)-4-phenyl-4H-1,2,4-triazol-3-yl)thio)acetate (**17d**)

Coarse white powder; yield, 70%; mp 232 °C; ^1^H NMR (DMSO-*d*_6_, 400 MHz) δ (ppm): 2.49 (s, 3H); 4.45 (s, 2H); 6.41 (s, 1H); 6.50 (s, 1H); 7.23 (d, *J* = 28 Hz 2H); 7.51–7.69 (m, 7 H); 7.87 (s, 2H). ^13^C NMR (DMSO-*d*_6_, 100 MHz): δ (ppm) 18.6, 34.5, 107.0, 110.2, 113.9, 114.3, 118.5, 118.5, 125.0, 125.0, 125.0, 127.1, 128.0, 128.1, 128.1, 129.3, 130.7, 133.3, 133.3, 144.3, 144.3, 144.5, 147.5, 153.0, 153.3, 153.3, 160.0, 167.4. MS *m*/*z*: 587.6 [M]^+^. Anal. Elem. Calc. for C_28_H_18_BrN_3_O_5_S: C, 57.15: H, 3.08: N, 7.14: Found: C, 57.17: H, 3.09: N, 7.17.

#### 3.2.5. 4-Methyl-2-oxo-2H-chromen-7-yl-2-((5-(5-bromobenzofuran-2-yl)-4-(4-methoxyphenyl)-4H-1,2,4-triazol-3-yl)thio)acetate (**17e**)

Coarse gray solid; yield, 62%; mp 220 °C ^1^H NMR (CDCl_3_, 400 MHz) δ (ppm): 2.40 (s, 3H); 3.91 (s, 3H); 4.34 (s, 2H); 6.25 (s, 1H); 6.42 (s, 1H); 7.08 (d, *J* = 8 Hz, 2H); 7.13 (s, 1H); 7.17 (d, *J* = 8 Hz, 1H); 7.31 (d, *J* = 8 Hz, 3H); 7.39 (d, *J* = 8 Hz, 1H); 7.59 (d, *J* = 8 Hz, 2H). ^13^C NMR (CDCl_3_, 100 MHz): δ (ppm) 18.7, 34.6, 55.7, 106.8, 110.2, 113.2, 113.2, 114.6, 115.4, 116.6, 118.0, 118.1, 124.2, 125.0, 125.5, 128.6, 129.1, 129.2, 143.6, 148.0, 148.0, 151.8, 152.7, 152.8, 153.5, 154.0, 160.3, 161.3, 166.4. MS *m*/*z*: 617.6 [M]^+^. Anal. Elem. Calc. for C_29_H_20_BrN_3_O_6_S: C, 56.62: H, 3.26: N, 6.79: Found: C, 56.65: H, 3.27: N, 6.80.

#### 3.2.6. 4-Methyl-2-oxo-2H-chromen-7-yl-2-((5-(5-chlorobenzofuran-2-yl)-4-phenyl-4H-1,2,4-triazol-3-yl)thio)acetate (**17f**)

Amorphous white solid; yield, 72%; mp 226 °C; ^1^H NMR (DMSO-*d*_6_, 400 MHz) δ (ppm): 2.49 (s, 3H); 4.45 (s, 2H); 6.41 (s, 1H); 6.50 (s, 1H); 7.23 (t, *J* = 28 Hz, 1H); 7.38 (d, *J* = 12 Hz, 2H); 7.57–7.85 (m, 7H); 7.88 (d, *J* = 12 Hz, 1H). ^13^C NMR (DMSO-*d*_6_, 100 MHz): δ (ppm) 18.6, 34.5, 107.1, 110.2, 113.4, 114.3, 114.3, 118.5, 122.0, 126.6, 126.6, 126.6, 127.1, 128.0, 128.5, 129.1, 129.1, 130.7, 131.4, 133.32, 144.5, 144.5, 147.5, 152.6, 152.6, 153.9, 160.0, 167.4. MS *m*/*z*: 543.9 [M]^+^. Anal. Elem. Calc. for C_28_H_18_ClN_3_O_5_S: C, 61.82: H, 3.34: N, 7.72: Found: C, 61.84: H, 3.35: N, 7.70.

#### 3.2.7. 4-Methyl-2-oxo-2H-chromen-7-yl2-((5-(5-chlorobenzofuran-2-yl)-4-(4-methoxyphenyl)-4H-1,2,4-triazol-3-yl)thio)acetate (**17g**)

Coarse off-white solid; yield, 67%; mp 200 °C; ^1^H NMR (CDCl_3_, 400 MHz) δ (ppm): 2.32 (s, 3H); 3.68–4.12 (m, 3H); 4.12 (s, 2H); 6.01 (s, 1H); 6.74 (t, *J* = 16 Hz, 2H); 6.83–6.92 (m, 1H); 7.07 (t, *J* = 16 Hz, 2H); 7.15–7.30 (m, 5H); 7.35 (t, *J* = 24 Hz 1H). ^13^C NMR (CDCl_3_, 100 MHz): δ (ppm) 18.6, 21.0, 22.6, 31.5, 55.4, 60.4, 103.3, 110.1, 110.7, 112.7, 113.5, 114.3, 115.4, 115.6, 118.0, 121.2, 125.6, 125.6, 126.5, 128.6, 128.9, 128.9, 152.8, 152.8, 153.4, 155.0, 160.9, 162.2, 171.3. MS *m*/*z*: 571.9 [M]^+^. Anal. Elem. Calc. for C_29_H_20_ClN_3_O_6_S: C, 60.68: H, 3.51: N, 7.32: Found: C, 60.71: H, 3.52: N, 7.34.

#### 3.2.8. 4-Methyl-2-oxo-2H-chromen-7-yl-2-((5-(naphtho[2,1-b]furan-2-yl)-4-phenyl-4H-1,2,4-triazol-3-yl)thio)acetate (**17h**)

Yellow solid; yield, 63%; mp 218 °C; ^1^H NMR (CDCl_3_, 400 MHz) δ (ppm): 2.35 (s, 3H); 4.32 (s, 2H); 6.19 (s, 1H); 7.08 (s, 1H); 7.14–7.21 (m, 3H); 7.42 (d, *J* = 8 Hz, 3H); 7.48 (t, *J* = 16 Hz 1H); 7.54–7.63 (m, 5H); 7.66–7.87 (m, 2H). ^13^C NMR (CDCl_3_, 100 MHz): δ (ppm) 18.7, 34.5, 110.2, 110.2, 112.2, 112.2, 114.6, 118.0, 122.8, 123.2, 125.7, 125.6, 126.9, 126.9, 127.8, 127.8, 127.8, 128.8, 128.8, 128.8, 128.8, 130.3, 130.4, 131.1, 132.9, 140.98, 151.8, 152.8, 152.8, 154.0, 160.4, 166.3. MS *m*/*z*: 559.9 [M]^+^. Anal. Elem. Calc. for C_32_H_21_ClN_3_O_5_S: C, 68.68: H, 3.78: N, 7.51: Found: C, 68.70: H, 3.76: N, 7.52.

### 3.3. Tyrosine Assay of Inhibitory Activity of Target Compounds

The synthesized coumarin–triazole hybrids were subjected to spectrophotometric assay for tyrosinase by employing Ellman’s method [55,56]. The tyrosine enzyme was extracted from *Bacillus subtilis* NCTC 10400. The anti-tyrosinase potential of all the coumarin–triazole hybrids was screened by using L-DOPA (levodopa) as the substrate. All the coumarin derivatives were dissolved in DMSO. A 2.0% solution of the test compound was made in DMSO, and the stock solution was further diluted by using phosphate buffer at pH 6.8. The coumarin derivatives along with the tyrosine enzyme (0.5 mg/mL) were pre-incubated for 10 min in buffer at 25 °C. Pre-incubation of tyrosinase and coumarin derivative was followed by the addition of levodopa (0.5 mM), and the reaction was monitored by absorbance measurement at 475 nm. The IC_50_ values were calculated by interpolation of the dose–response curves. The following formula was applied to calculate the percentage inhibition of tyrosinase:

%age Inhibition = (B − S/B) × 100

where S and B are the absorbance values for the sample and blank, respectively. For comparison, we used ascorbic acid as the reference inhibitor.

### 3.4. In Silico Modeling Method

For IFD (induced fit docking), the most potent compound, **17e**, was chosen. The Molecular Operating Environment (MOE 2015) [57] was used for all docking operations. The desired compound (**17e**) was produced by using the ChemDraw program. In the IFD simulation, Kojic acid was used as the standard, and its 3D conformer (CID: 3840) was taken from PubChem. The Protein Data Bank (PDB) [58] provided the crystalline structure of tyrosinase (2Y9X), which was utilized as the receptor for use in IFD. The MOE Quick-Prep tool was utilized for preparing the molecular structures of the ligand and protein. The docking system’s energy consumption was reduced by using the standard Amber10:EHT forcefield with a gradient of 0.1. The following methods were utilized for assessment: London-dG, WSA/GBVI scoring, Induced Fit refinement, and Triangular Matcher placement. To verify the docking approach, cognate redock was applied to the native ligand. The analysis of the ligand–protein interactions was carried out in BIOVIA Discovery Studio 2021.

## 4. Conclusions

A library of coumarin–triazole hybrids was synthesized by the coupling of different benzofuran-based triazoles with 7-hydroxy-4-methylcoumarin. The synthesized coumarin derivatives with various electron-donating and electron-withdrawing groups were characterized by various analytical techniques. The anti-tyrosinase activity of the synthesized coumarin–triazole hybrids was assessed through in vitro screening. A computational study was also carried out to verify the findings interpreted from the in vitro screening of anti-tyrosinase activity. All the synthesized compounds were found more active against the tyrosinase enzyme than Kojic acid and ascorbic acid (standards). The in vitro study indicated that in the library of derived compounds, **17e** showed excellent anti-tyrosinase activity, with an IC_50_ = 0.339 ± 0.08. Moreover, **17f** exhibited remarkable potency, with an IC_50_ of 3.148 ± 0.23 μM, showing that the presence of EDG on the aromatic ring of the synthesized compounds improved anti-tyrosinase activity compared with compounds with unsubstituted aromatic rings. Therefore, the presence of EWG (Br and Cl) on the benzofuran moiety of the coumarin scaffold appears to modify the pharmacological behavior of these coumarin–triazole hybrids. The mode of action of compounds **17e** and **17f** against tyrosinase was determined by docking studies, and the results of the docking analysis of the mentioned compounds were in accordance with the biological diagnostic results, confirming that these hybrids could be established as lead compounds.

## Figures and Tables

**Table 1:** Inhibitory activities of title compounds **17a**–**h** against tyrosinase.

Sr. No.	Target Compound	R^1^	R^2^	%Age Inhibition	IC_50_ (µM) ^a^
1	**17a**	H	C_6_H_5_	12.02 ± 0.21	8.13 ± 0.13
2	**17b**	7-OMe	C_6_H_5_	6.30 ± 0.14	14.06 ± 0.17
3	**17c**	7-OMe	4-OMeC_6_H_4_	5.11 ± 0.15	12.98 ± 0.32
4	**17d**	5-Br	C_6_H_5_	23.09 ± 0.13	5.89 ± 0.09
5	**17e**	5-Br	4-OMeC_6_H_4_	30.85 ± 0.15	0.33 ± 0.08
6	**17f**	5-Cl	C_6_H_5_	36.13 ± 0.21	3.14 ± 0.23
7	**17g**	5-Cl	4-OMeC_6_H_4_	31.060 ± 0.19	3.98 ± 0.12
8	**17h**	Fused C_6_H_4_	C_6_H_5_	30.62 ± 0.33	5.13 ± 0.05
10	Ascorbic acid (standard)			58.66 ± 1.00	11.5 ± 1.00
11	Kojic acid (standard)			6.79 ± 0.58	30.34 ± 0.75

^a^ Values are given as means ± SEs (n = 3).

**Table 2:** Ligand–receptor interaction study.

Interaction Study	Kojic Acid	Compound 17e	Compound 17f
**Docking score (kcal/mol)**	−5.25	−6.75	−6.29
**Ligand–receptor interaction**	VAL-283,ALA-286, GLY-281, HIS-61, HIS-263, HIS-85, and MET-280	GLY-281, HIS-263, HIS-259, VAL-283, HIS-85, PHE-264, and VAL-248	VAL-268, LEU-275, VAL-283, HIS-85, HIS-259, HIS-263, GLY-281, and ARG-268
**Interaction types**	π–π stacked, hydrogen bonding, and π–alkyl	C-H bond, alkyl, π–π T-shaped, and alkyl–π	Carbon–hydrogen bond, alkyl, π–alkyl, and π–donor hydrogen bond

## Data Availability

All the data are contained within the article and the Supplementary Materials.

## Supplementary Materials

The following supporting information can be downloaded at: https://www.mdpi.com/article/10.3390/ph17040532/s1, Figures S1–S16: ^1^H and ^13^C NMR spectra of compounds **17a**–**h**.
