# Supplementary material for: Synergistic Biomedical Potential and Molecular Docking Analyses of Coumarin–Triazole Hybrids as Tyrosinase Inhibitors: Design, Synthesis, In Vitro Profiling, and In Silico Studies
Source: Pharmaceuticals (Basel). 2024 Apr 20;17(4):532. doi: 10.3390/ph17040532 (PMC11054269; doi:10.3390/ph17040532)
Supplement: Supplementary file 1 [file pharmaceuticals-17-00532-s001.zip › pharmaceuticals-2955173-supplementary.pdf]

Supporting Information for

Synergistic Biomedical Potential and Molecular Docking Analysis of Coumarin-Triazole Hybrids as Tyrosinase Inhibitors: Design, Synthesis, *In Vitro* Profiling and *In Silico* Studies

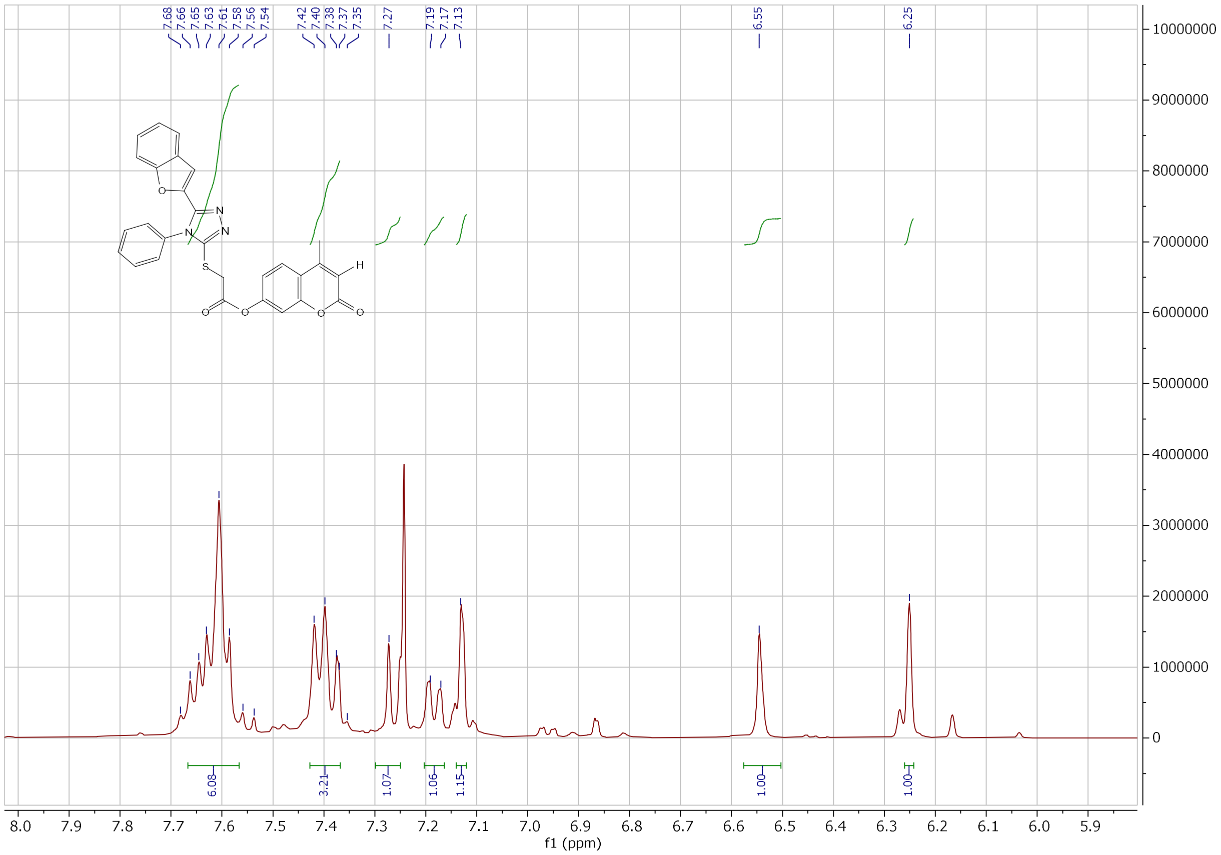

Figure S1: ^1H NMR (400 MHz, $CDCl_3$) of **17a**

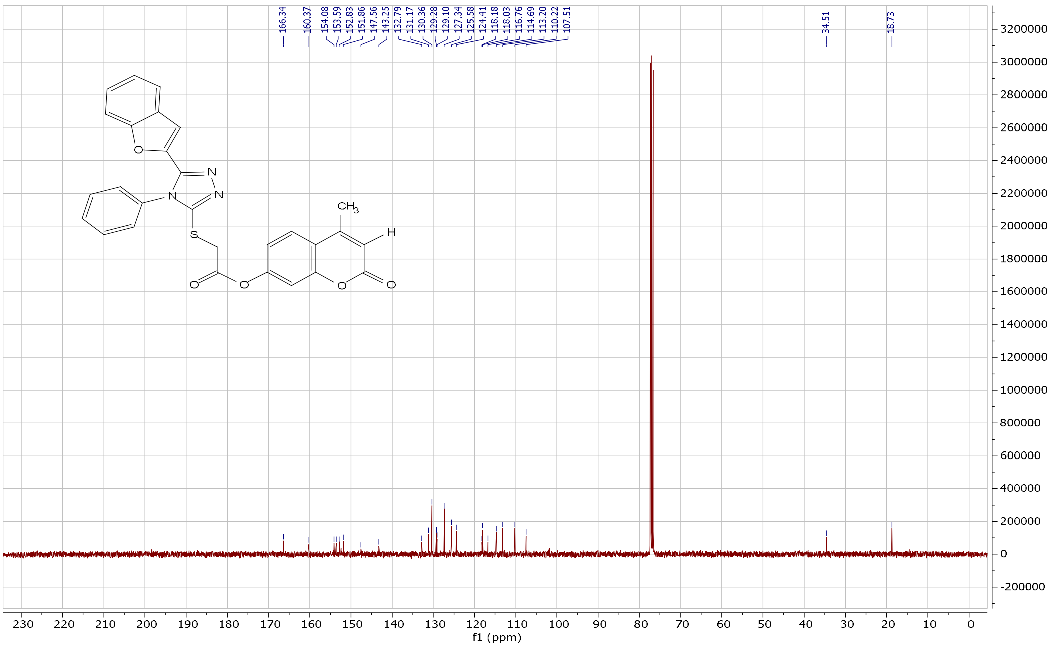

Figure S2: ^{13}C NMR (100 MHz, $CDCl_3$) of **17a**

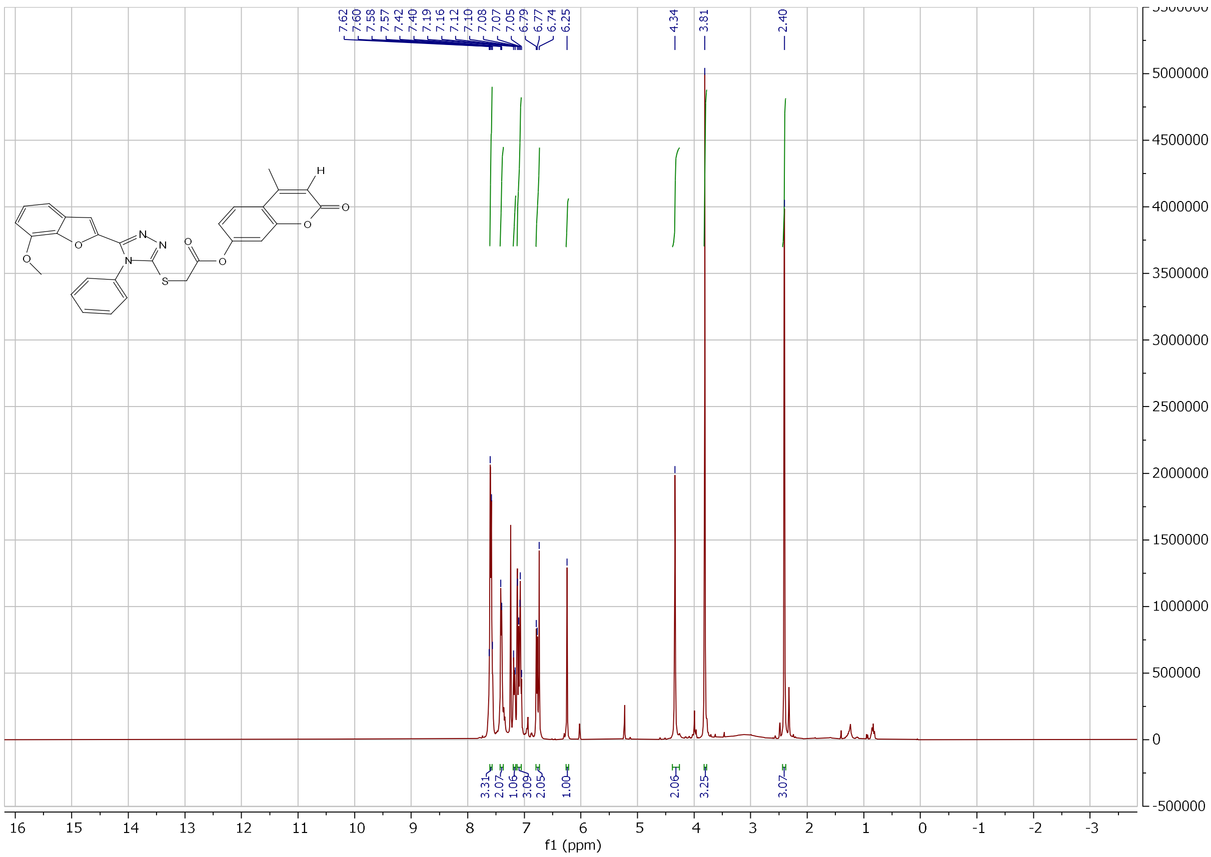

Figure S3: ^{1}H NMR (400 MHz, $CDCl_3$) of **17b**

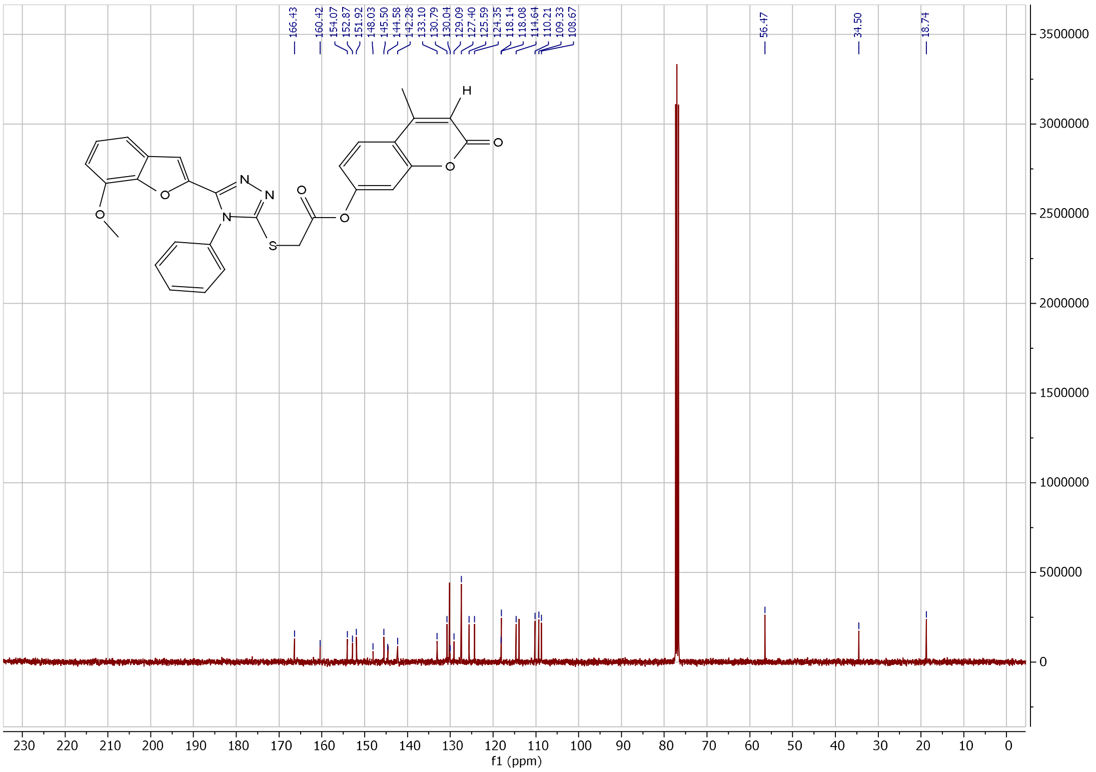

Figure S4: ^{13}C NMR (100 MHz, $CDCl_3$) of **17b**

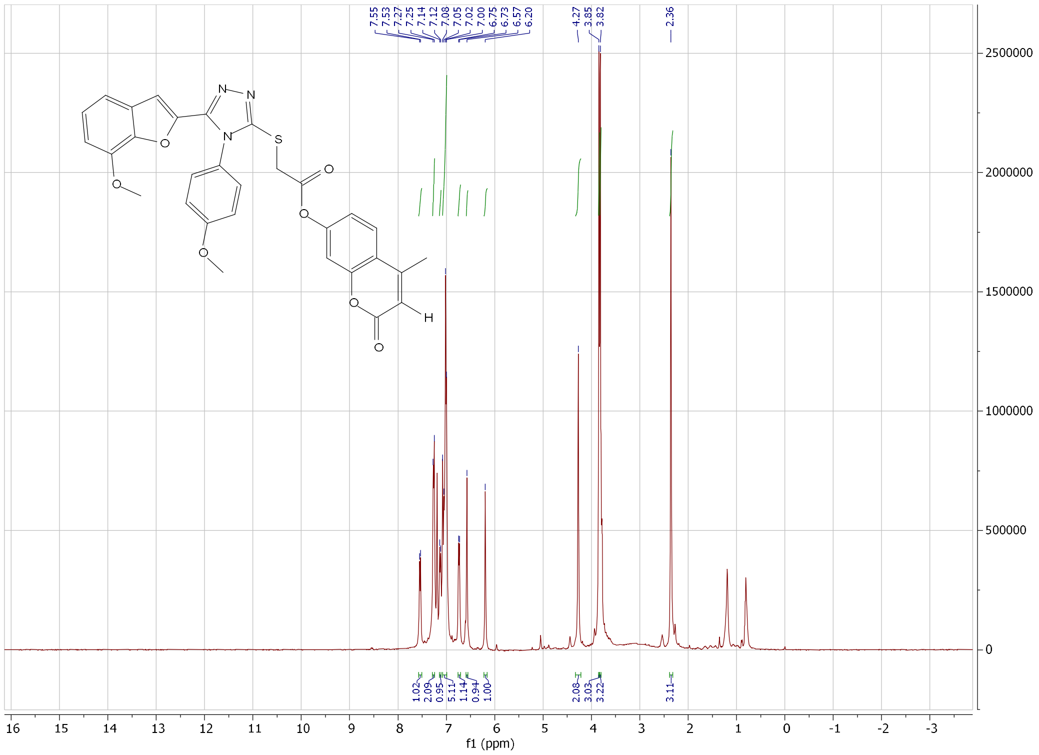

Figure S5: ^{1}H NMR (400 MHz, $CDCl_3$) of **17c**

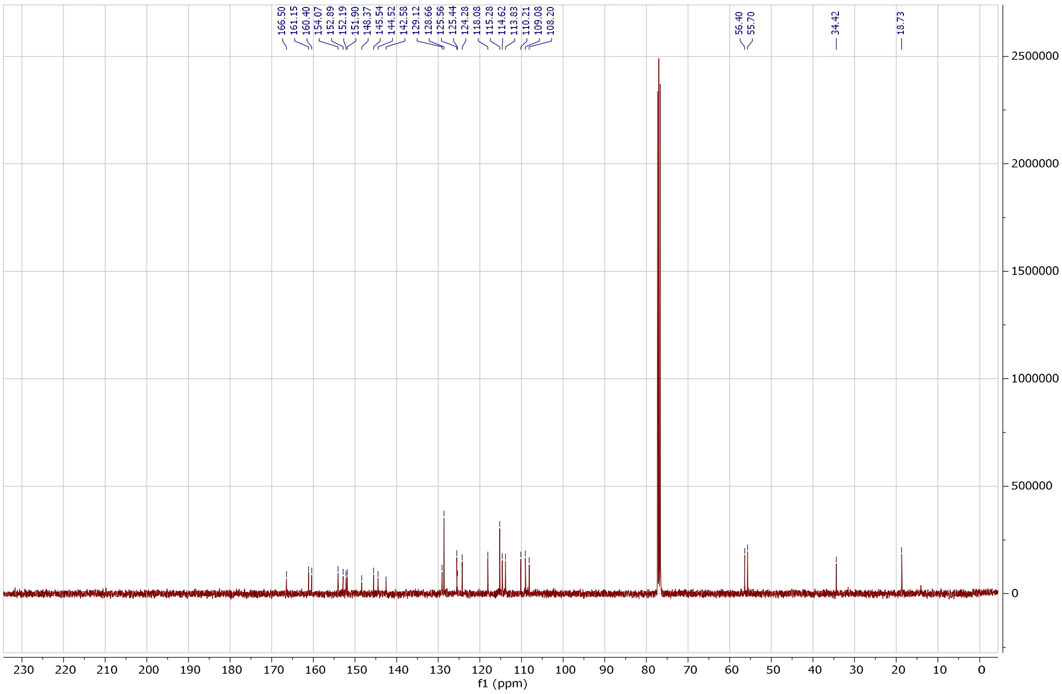

Figure S6: ^{13}C NMR (100 MHz, $CDCl_3$) of **17c**

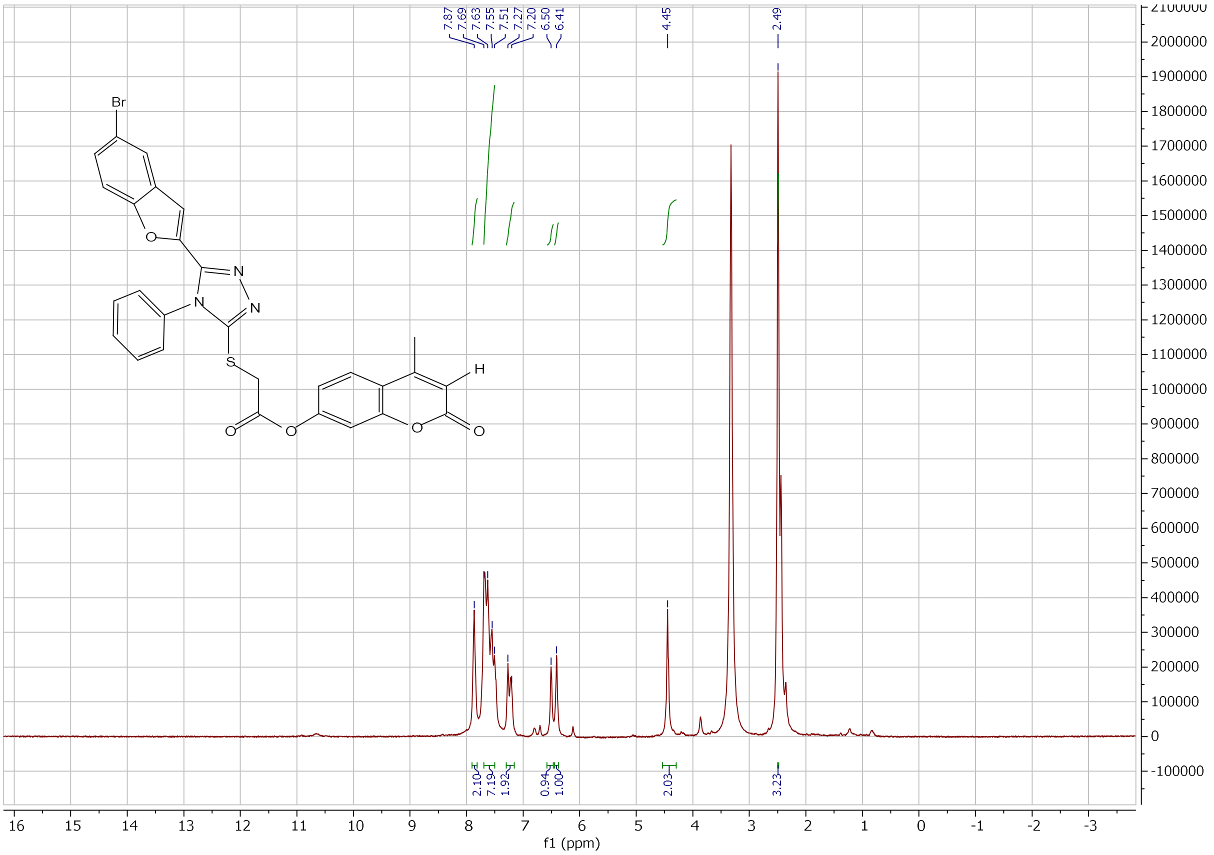

Figure S7: ^{1}H NMR (400 MHz, $CDCl_3$) of **17d**

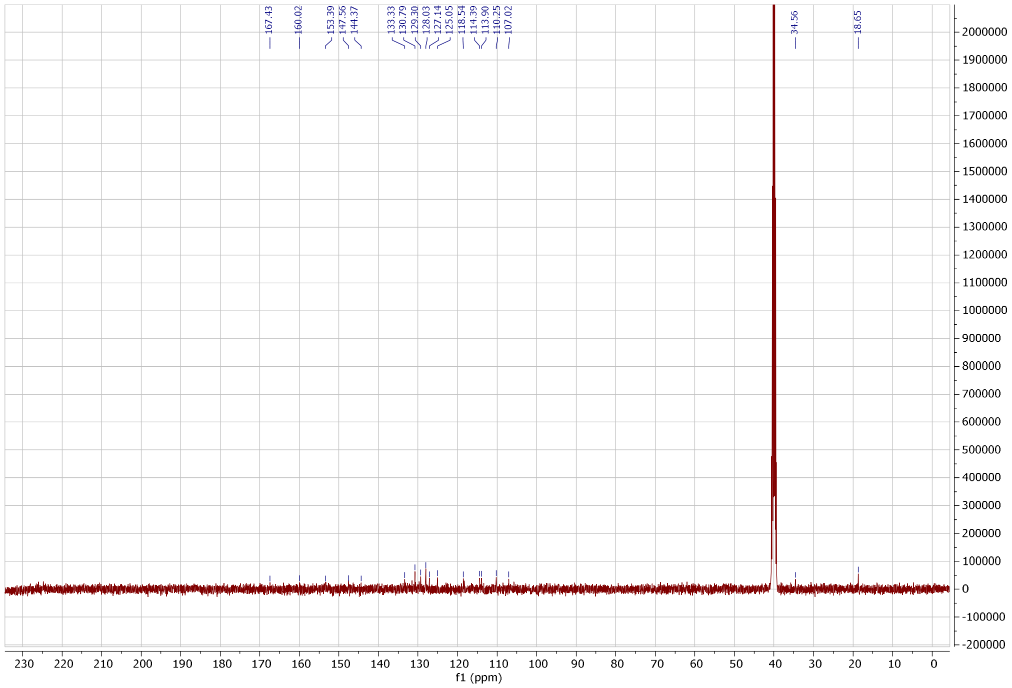

Figure S8: ^{13}C NMR (100 MHz, $CDCl_3$) of **17d**

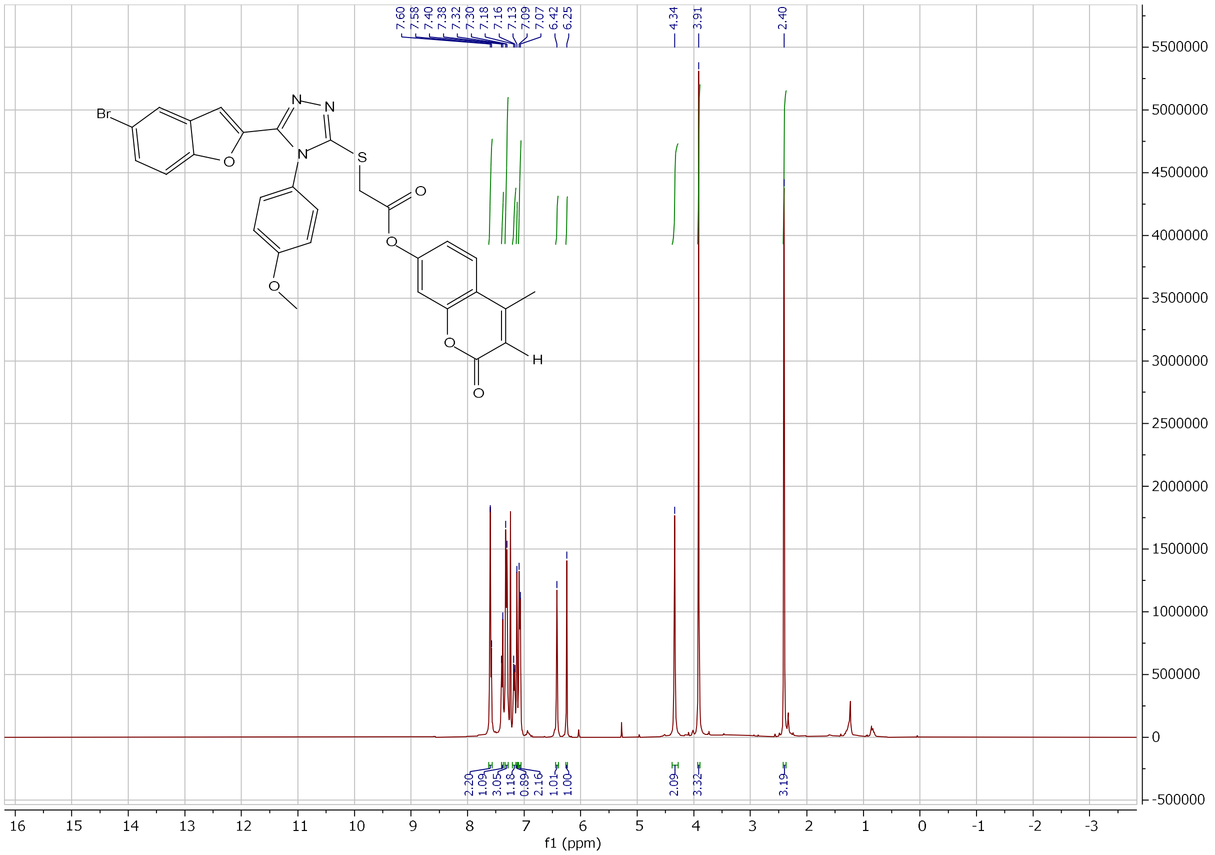

Figure S9: ^{1}H NMR (400 MHz, $CDCl_3$) of **17e**

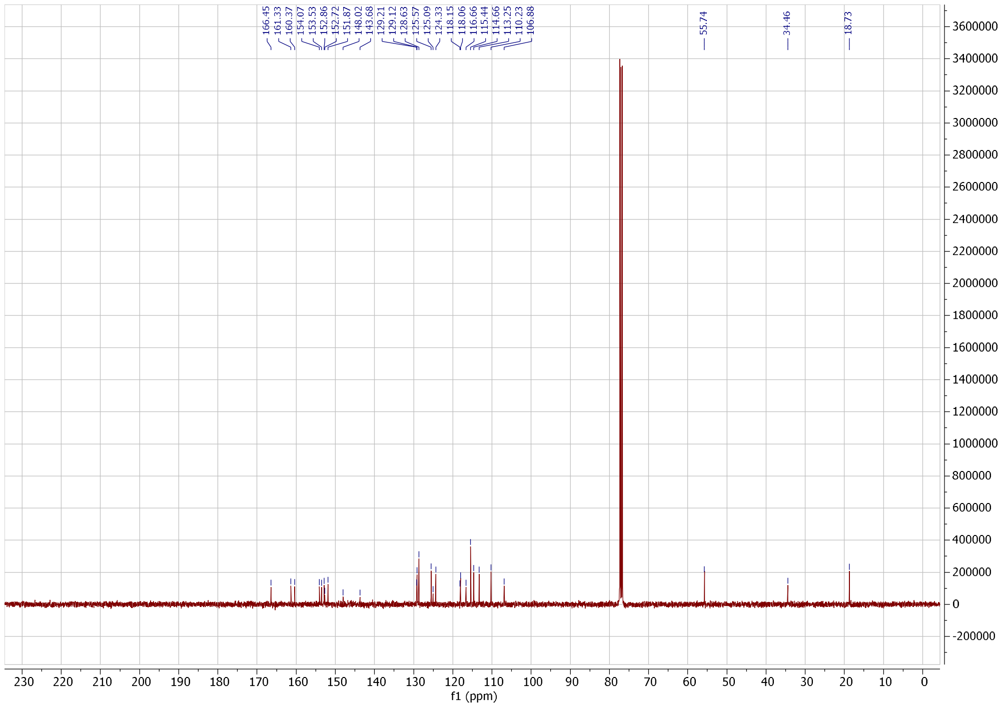

Figure S10: ^{13}C NMR (100 MHz, $CDCl_3$) of **17e**

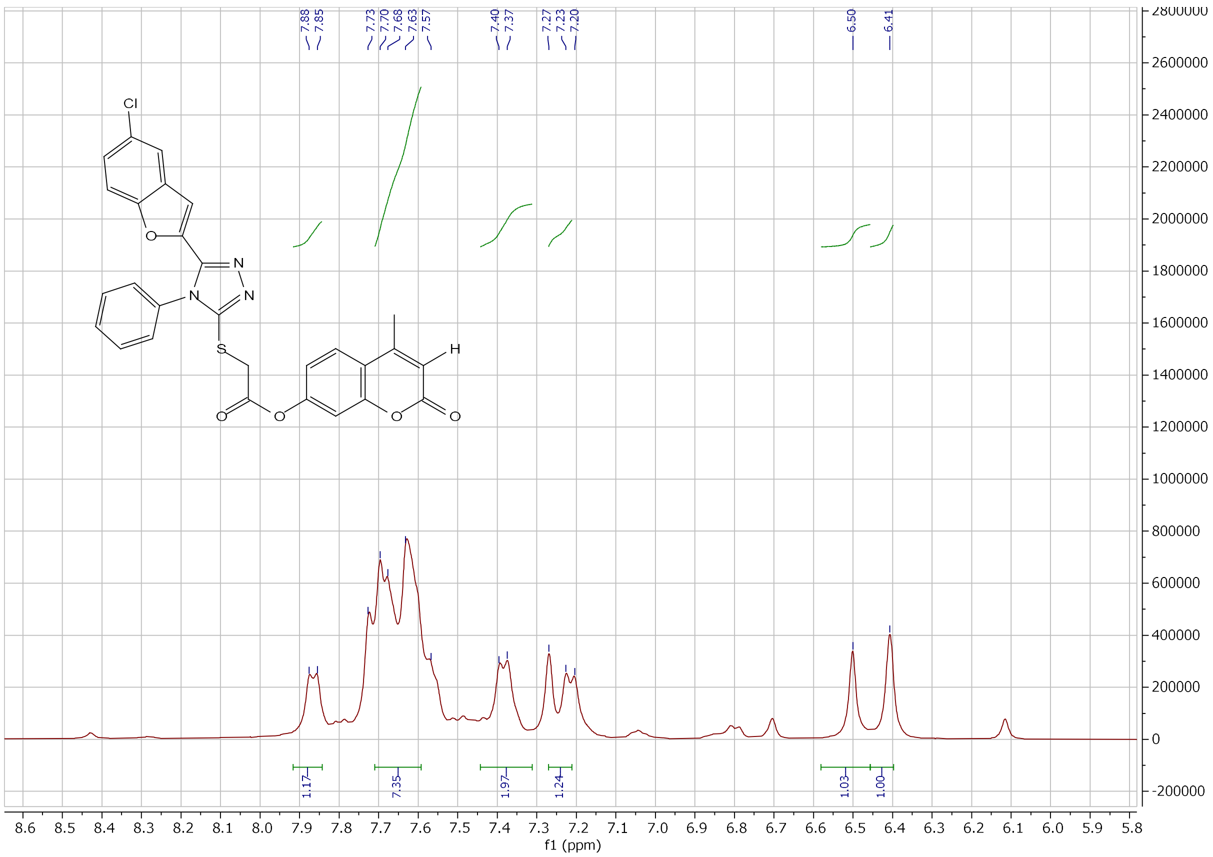

Figure S11: ^{1}H NMR (400 MHz, $CDCl_3$) of **17f**

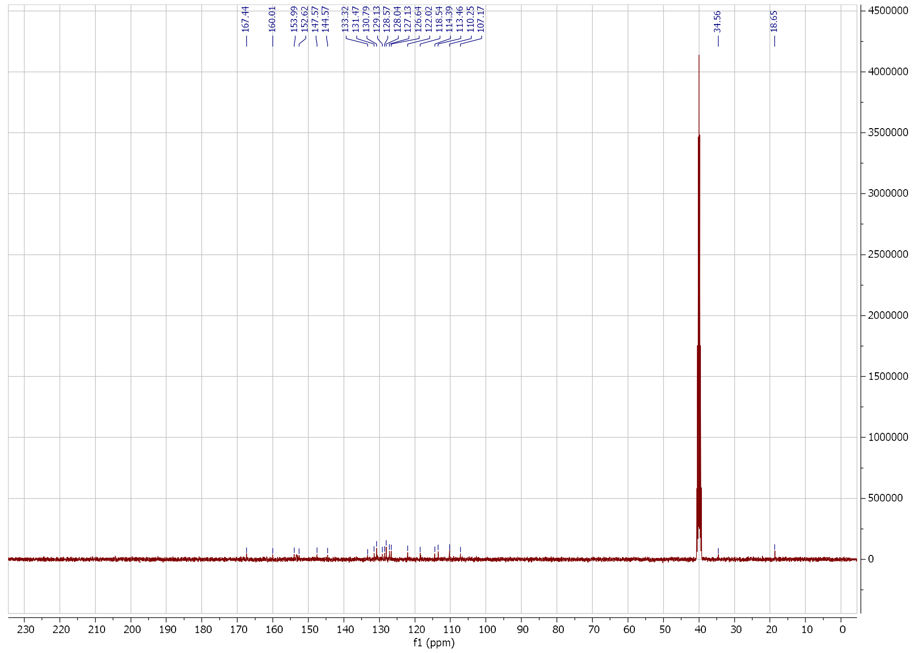

Figure S12: ^{13}C NMR (100 MHz, $CDCl_3$) of **17f**

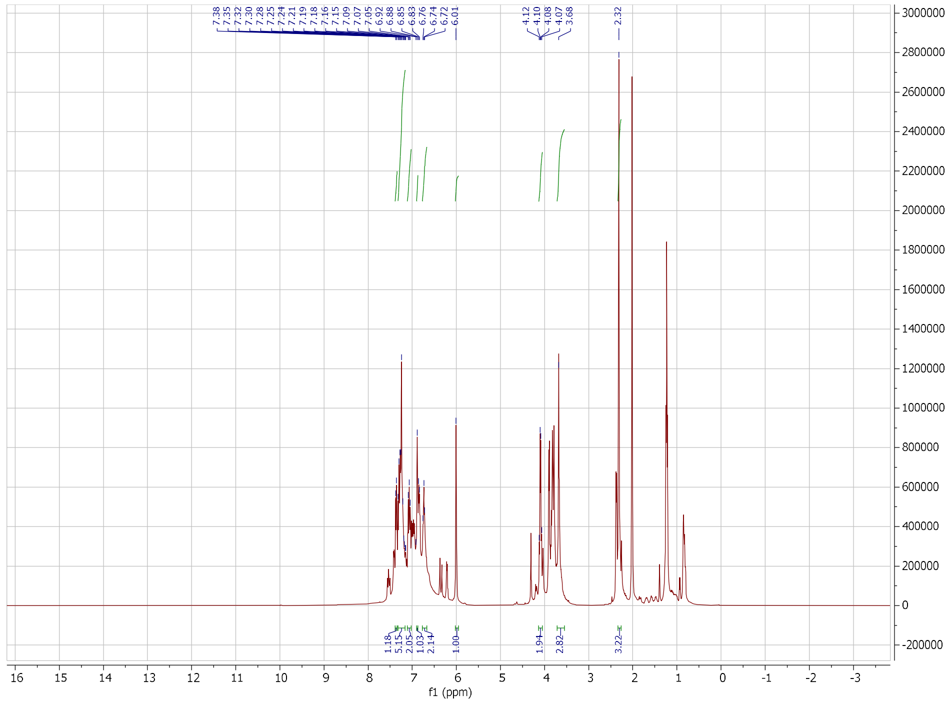

Figure S13: ^{1}H NMR (400 MHz, $CDCl_3$) of **17g**

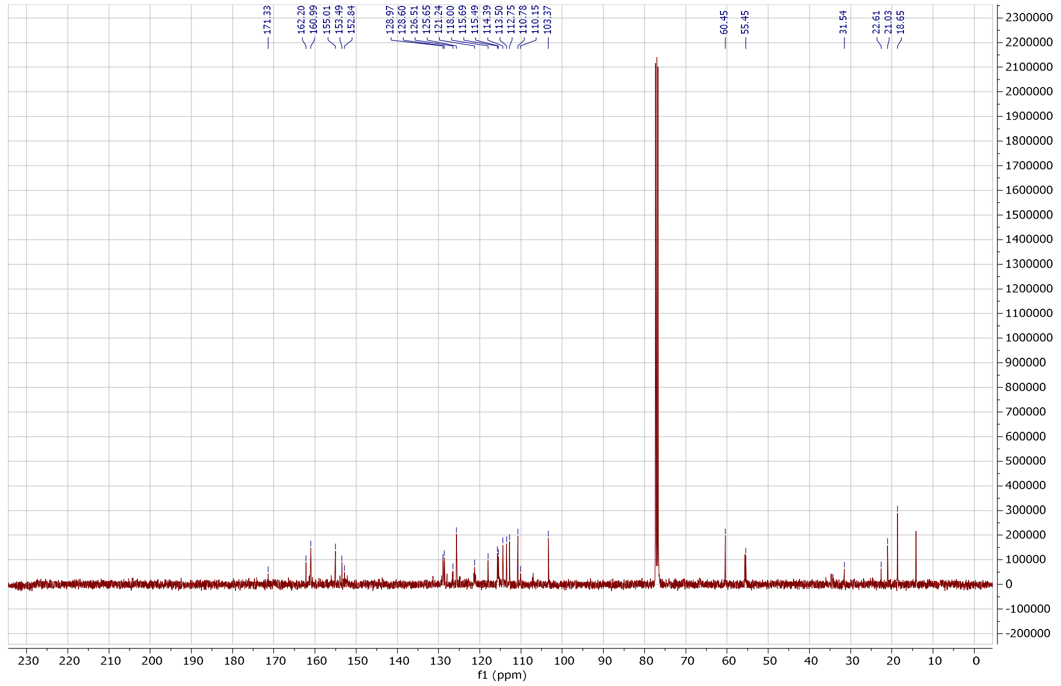

Figure S14: ^{13}C NMR (100 MHz, $CDCl_3$) of **17g**

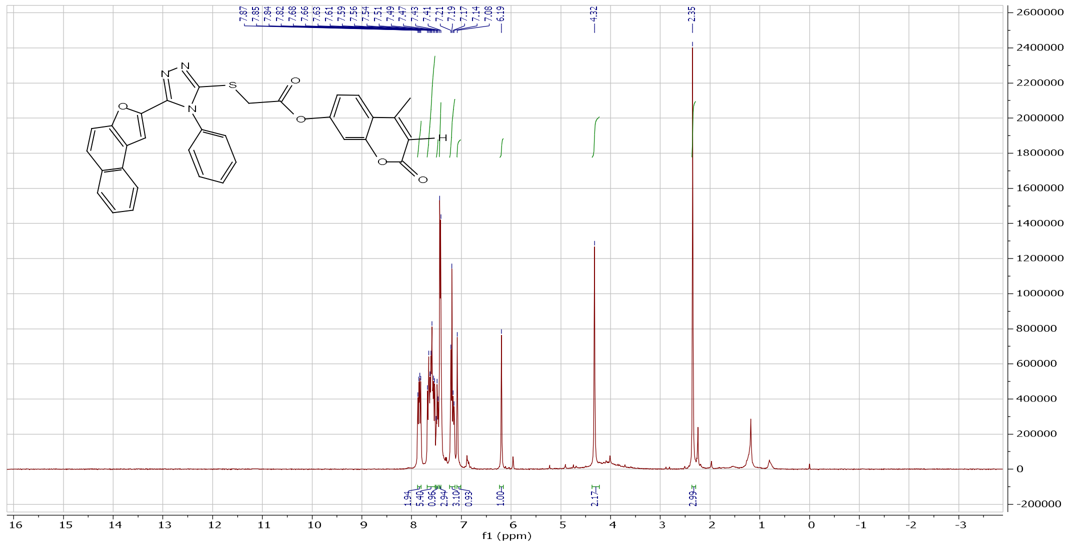

Figure S15: ^{1}H NMR (400 MHz, $CDCl_3$) of **17h**

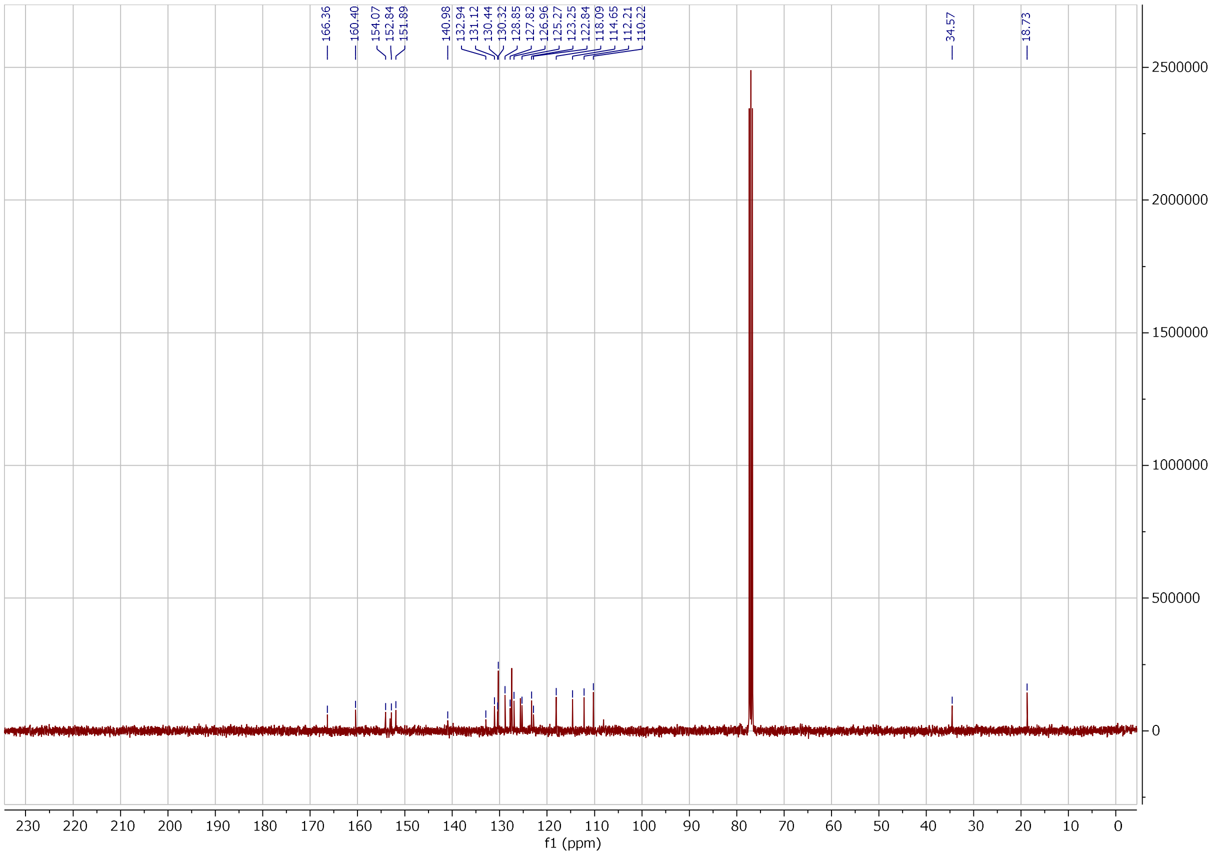

Figure S16: ^{13}C NMR (100 MHz, $CDCl_3$) of **17h**
